# The genetic network of greater sage‐grouse: Range‐wide identification of keystone hubs of connectivity

**DOI:** 10.1002/ece3.4056

**Published:** 2018-05-04

**Authors:** Todd B. Cross, Michael K. Schwartz, David E. Naugle, Brad C. Fedy, Jeffrey R. Row, Sara J. Oyler‐McCance

**Affiliations:** ^1^ USDA Forest Service National Genomics Center for Wildlife and Fish Conservation Rocky Mountain Research Station Missoula Montana; ^2^ College of Forestry and Conservation University of Montana Missoula Montana; ^3^ School of Environment, Resources and Sustainability University of Waterloo Waterloo ON Canada; ^4^ U.S. Geological Survey Fort Collins Science Center Fort Collins Colorado

**Keywords:** *Centrocercus urophasianus*, graph theory, multiscale conservation prioritization

## Abstract

Genetic networks can characterize complex genetic relationships among groups of individuals, which can be used to rank nodes most important to the overall connectivity of the system. Ranking allows scarce resources to be guided toward nodes integral to connectivity. The greater sage‐grouse (*Centrocercus urophasianus*) is a species of conservation concern that breeds on spatially discrete leks that must remain connected by genetic exchange for population persistence. We genotyped 5,950 individuals from 1,200 greater sage‐grouse leks distributed across the entire species’ geographic range. We found a small‐world network composed of 458 nodes connected by 14,481 edges. This network was composed of hubs—that is, nodes facilitating gene flow across the network—and spokes—that is, nodes where connectivity is served by hubs. It is within these hubs that the greatest genetic diversity was housed. Using indices of network centrality, we identified hub nodes of greatest conservation importance. We also identified keystone nodes with elevated centrality despite low local population size. Hub and keystone nodes were found across the entire species’ contiguous range, although nodes with elevated importance to network‐wide connectivity were found more central: especially in northeastern, central, and southwestern Wyoming and eastern Idaho. Nodes among which genes are most readily exchanged were mostly located in Montana and northern Wyoming, as well as Utah and eastern Nevada. The loss of hub or keystone nodes could lead to the disintegration of the network into smaller, isolated subnetworks. Protecting both hub nodes and keystone nodes will conserve genetic diversity and should maintain network connections to ensure a resilient and viable population over time. Our analysis shows that network models can be used to model gene flow, offering insights into its pattern and process, with application to prioritizing landscapes for conservation.

## 1 INTRODUCTION

Understanding population structure and quantifying genetic connectivity are important for guiding ongoing conservation and restoration efforts (Crooks & Sanjayan, 2006). Traditionally, population structure is first analyzed and subpopulations delineated, then genetic connectivity among subpopulations is quantified. However, this process need not be completed in two stages. Genetic network models can be used to simultaneously gain an understanding of population structure and to quantify genetic connectivity among populations in natural systems (Bunn, Urban, & Keitt, 2000; Dyer, 2007; Dyer & Nason, 2004).

Genetic networks are constructed of components called nodes and edges, where nodes may represent populations and edges represent genetic connectivity among nodes (Sallaberry, Zaidi, & Melançon, 2013). Each node can be weighted by the genetic diversity within the nodes and each edge by the genetic covariance among local populations (Bunn et al., 2000). The overall structure of the network provides a means by which to rank the importance of how each component contributes to maintaining network connectivity (Jacoby & Freeman, 2016). One can think of network structure in terms of the commercial airline model. In such models, nodes and edges are known, as are nodes of high and low connectivity (henceforth, hub nodes and spoke nodes, respectively). In the airline industry, hub nodes are strategically selected to maximize efficiency of air traffic, while spoke nodes are selected based on limited need for services. For wildlife populations, where populations serve as hub nodes and where populations serve as spoke nodes are unknown.

Qualifying genetic network structure and identifying nodes that act as hubs can be very informative to conservation and management of wildlife species (Garroway, Bowman, Carr, & Wilson, 2008; Lookingbill, Gardner, Ferrari, & Keller, 2010). Knowledge of which nodes are connected to one another and which nodes rank highly in network centrality can facilitate prioritization for management (Jacoby & Freeman, 2016). Prior network modeling of wildlife populations have shown that which nodes function as hubs and which function as spokes is not intuitive (Bunn et al., 2000; Garroway, Bowman, & Wilson, 2011; Garroway et al., 2008; Koen, Bowman, & Wilson, 2015). One might expect a node's proximity to the center of the species’ range would influence that node's importance to connectivity, where centrally located nodes have greater genetic exchange than peripheral nodes. However, it has been shown that populations at the periphery of a species’ range can act as critical hub nodes, connecting populations across the network, and that populations located toward the center of the range do not necessarily function as hub nodes (Bunn et al., 2000).

Emergent properties of genetic networks can be used to identify hub nodes and spoke nodes and the sensitivity of the entire network to the loss of connectivity (Dunne, Williams, & Martinez, 2002). There are three common network structures: (1) single‐scale (“regular”), (2) broad‐scale (“random”), and (3) small‐world—a subset of which are known as scale‐free (Amaral, Scala, Barthélémy, & Stanley, 2000; Bray, 2003). Regular networks are highly structured such that proximal nodes tend to be linked to each other, while distant nodes tend not to be linked: a structure comparable to the isolation by distance pattern commonly discovered in the population genetics literature (e.g., a stepping‐stone model; Wright, 1943). In a regular genetic network, genetic connectivity is between neighboring nodes and nodes separated by a greater number of edges will be more isolated from one another. In regular networks, hub nodes are nonexistent as all nodes are equally connected. Random networks are unstructured such that proximity of nodes is irrelevant to whether nodes are connected or not and to the strength of connections: a structure most similar to the theoretical island model first proposed by Wright (1931) and analogous to the population genetic concept of panmixia. In a random genetic network, genetic connectivity is unencumbered across the entire network because the number of steps between any two nodes is relatively small such that close and distant nodes have equal chances of being linked. In random networks, there are no hub nodes, but there exist thoroughfares through the network that foster quick transit among any set of nodes. In contrast, small‐world networks are composed of few highly connected nodes (hub nodes) and a greater number of more isolated nodes (spoke nodes), much like the hub‐and‐node model characteristic of the familiar commercial airline model. Most nodes can be reached from every other node by a small number of steps, often routed through central hub nodes, which foster connectivity among the spoke nodes. Redundancy is an important characteristic of small‐world networks. In small‐world genetic networks, genetic connectivity is greatest among nearest neighbor nodes, but genetic connectivity can exist between any two nodes by a small number of steps through hub nodes which are nodes at which genetic connectivity is concentrated such that these nodes serve to connect other distal nodes (also known as, spoke nodes). An extreme form of small‐world networks is scale‐free networks. In scale‐free networks, there is less redundancy in internode connections and greater centrality for the hub nodes.

Within any network's structure, individual node importance to network connectivity can be quantified by centrality indices. There are several centrality indices, each of which quantifies the importance of a node to network connectivity in a different way (Table 1). Some centrality indices rank nodes based on local connectivity and some based on network‐wide connectivity. Therefore, these function‐valued centrality indices can be easily transformed into node‐specific rankings, and these rankings can be used to prioritize conservation (Jacoby & Freeman, 2016).

**Table 1:** Network parameters used to quantify connectivity, the unit for which each is calculated, and the definition of the parameter, and relation of the parameter as pertains to the greater sage‐grouse population network. All but characteristic path length and weight are centrality indices

Network parameter	Network unit	Definition source	Ecological interpretation
Characteristic path length	Entire network	The mean of all pairwise network distances connecting nodes^b^	Mean number of steps for genetic exchange among all nodes along all possible paths
Betweenness centrality	Node	The number of shortest paths upon which a particular node lies^b^	The importance of node to maintaining network‐wide genetic exchange along the most direct routes
Closeness centrality	Node	The mean shortest path between node and all other nodes (connected network)	Mean number of steps for the most direct path of genetic exchange between any two nodes
Clustering coefficient	Node	The probability that two nodes connected to a given node are also connected (ranges from 0–1)^b^	An index of genetic connectivity among nodes that are both connected to another node
Degree centrality	Node	The number of edges connected to a node^b^	The number of other nodes with which a given node exchanges genes
Eigenvector centrality	Node	The direct and indirect connectivity: per node and immediate neighbors^a^	An index of how well connected a given nodes’ connections are as follows: that is, how much genetic exchange occurs at a node's immediate connections
Strength	Node	The sum of all edge weights^b^	An index of the magnitude of genetic exchange with all nodes connected to a given node
Weight	Edge	The magnitude of covariance between connected nodes	The magnitude of genetic exchange between any two connected nodes

a Garroway et al. (2008).

b Newman (2006).

**Figure 1:**
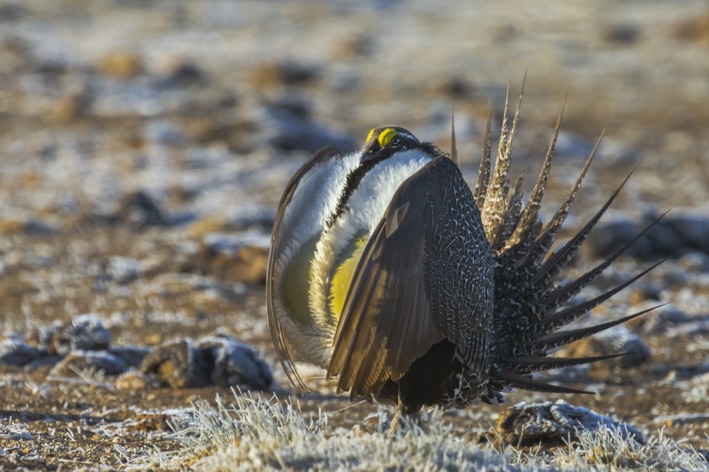
A male greater sage‐grouse (*Centrocercus urophasianus*) displays on a lek in the early morning. Photograph credit: Rick McEwan

The greater sage‐grouse (*Centrocercus urophasianus*; hereafter, sage‐grouse; Figure 1) is a lekking gallinaceous bird of conservation concern, an indicator species for sagebrush (*Artemisia* spp.) communities (Rowland, Wisdom, Suring, & Meinke, 2006), and an indicator species for landscape‐scale connectivity across the western United States and southern Canada (Aldridge et al., 2008). Sage‐grouse once occupied over 1.2 million km^2^ (Edminster, 1954; Schroeder et al., 2004). The species now occupies less than 0.67 million km^2^ across 11 western states and two Canadian provinces (Patterson, 1952; Schroeder et al., 2004)—56% of its range compared to pre‐Western Settlement (Schroeder et al., 2004). An additional 29% of the remaining species’ range is likely at risk of extirpation (Aldridge et al., 2008). Increased geographic isolation and declines in sage‐grouse populations range‐wide coincide with fragmentation and loss of sagebrush due to changes in land use (Copeland, Doherty, Naugle, Pocewicz, & Kiesecker, 2009; Knick et al., 2003; Schrag, Konrad, Miller, Walker, & Forrest, 2011).

Sagebrush provides essential cover, is a staple of the species’ diet, is where the bird nests and rears its broods, and congregates in the spring to display and breed on leks (Beever & Aldridge, 2011; Hagen, Connelly, & Schroeder, 2007; Patterson, 1952; Remington & Braun, 1985; Wallestad & Eng, 1975). On leks, males battle with one another to claim the center and energetically display to potential mates. Lek attendance by males is significantly correlated with female lek attendance (Bradbury, Vehrencamp, & Gibson, 1989).

Despite long seasonal migratory movements (up to 240 km; Smith, 2012) and large home ranges (4–195 km^2^; Connelly, Hagen, & Schroeder, 2011; Connelly, Rinkes, & Braun, 2011), fidelity to leks and stability in lek location are well documented (Cross, Naugle, Carlson, & Schwartz, 2017; Dalke, Pyrah, Stanton, Crawford, & Schlatterer, 1963; Dunn & Braun, 1985; Emmons & Braun, 1984; Patterson, 1952; Schroeder & Robb, 2003; Wallestad & Schladweiler, 1974). However, sage‐grouse may shift or abandon leks because of persistent disturbance or alteration of sagebrush cover (Holloran, Kaiser, & Hubert, 2010; Walker, Naugle, & Doherty, 2007).

Sage‐grouse are known to disperse during the breeding season and are capable of long‐distance breeding dispersal movements (Cross et al., 2017). While the fashion of long‐distance dispersal movements is unknown, most migratory movements are made in stepping‐stone fashion (Tack, 2009), and short‐distance abrupt singular movements are common when suitable habitat is lacking (Dunn & Braun, 1986).

The lek mating system of sage‐grouse is well suited to network analyses because leks are fairly fixed spatial locations. Given the species’ patterns of dispersal, we would expect that network structure should be composed of clustered, hub node‐like nodes characteristic of a small‐world network (Garroway et al., 2008).

New connectivity insights would come at a critical time for sage‐grouse. In 2010, sage‐grouse were added to the federal Endangered Species Act (ESA) candidate list following several petitions for protection (U.S. Fish and Wildlife Service 2010). In September 2015, a U.S. Fish and Wildlife Service determination found current efforts by state and federal agencies and other partners adequate to obviate the need for listing. However, significant conservation challenges remain, and the species’ status will again be reviewed in 2020 (U.S. Fish and Wildlife Service 2015). Understanding genetic connectivity is a critical step toward comprehending the relationship between the distribution and abundance of extant populations across fragmented landscapes. Perhaps more importantly, network analysis will greatly benefit planning in identifying conservation targets with the greatest benefit to maintaining genetic connectivity.

In this study, we had two primary objectives. First, we sought to determine the network structure of connectivity among leks, weighting edges by genetic divergence based on genetic covariance among leks. Second, we sought to identify which leks were important to maintaining overall population connectivity and persistence using network centrality indices. Within this second objective, we also sought to identify keystone nodes, that is, nodes that act as more important to maintaining gene flow than their size or location within the species range alone would indicate.

## 2 METHODS

### 2.1 Study area and sampling

We used 16,420 spatially referenced sage‐grouse feather and blood samples collected from 2,139 leks (mean of 7.68 samples per lek) across the entire contiguous range of the species in the United States of America and Canada from 2005 to 2015. The spatial distribution of our sampling was optimized as described in Hanks et al. (2016), following a smaller pilot sample. Feather samples were collected from leks using noninvasive methods (Bush, Vinsky, Aldridge, & Paszkowski, 2005; Segelbacher, 2002) after having been dropped by sage‐grouse during breeding activity, while blood samples were collected from sage‐grouse on leks as part of radiotelemetry field research. The only location throughout the entire distribution of the species that we did not use was Washington State because samples from this location were collected during a different period (from 1992 to 1999) than the rest of the samples.

### 2.2 DNA extraction

Genetic analysis was conducted at two molecular biological laboratories: the National Genomics Center for Wildlife and Fish Conservation at the USFS Rocky Mountain Research Station and the Molecular Ecology Lab at the USGS Fort Collins Science Center. Protocols were established at the inception of the study to ensure consistency among laboratory genotyping and are described below. Feather DNA was extracted from the quill (calamus) using QIAGEN DNeasy Blood and Tissue Kit and the user‐developed protocol for purification of total DNA from nails, hair, or feathers. The protocol was modified by incubating samples for a minimum of 8 h after addition of Proteinase K to maximize tissue lysis and by eluting DNA with 100 μl of Buffer AE to increase the final DNA concentration in the eluate. Blood samples were extracted using QIAGEN DNeasy Blood and Tissue Kit and the protocol for nucleated blood. At USGS, parts of the extraction process were automated using a QIAcube (Qiagen).

### 2.3 Microsatellite DNA amplification and genotyping

We based our analysis upon a panel of neutral, polymorphic microsatellite loci both to identify individuals from noninvasively collected samples (of unknown individual origin) and to quantify relatedness (i.e., functional movement resulting in gene flow). We amplified 15 variable microsatellite loci (BG6, SGMS064, SGMS066, SGMS068, MSP11, MSP18, SG28, SG29, SG36, SG39, SGCA5, SGCA11, SGCTAT1, TUT3, and TUT4) and one sex‐diagnostic locus [CHD gene, using the primers 1237L and 1272H (Kahn, St. John, & Quinn, 1998)]. Primer design, PCR conditions, and electrophoresis used at USFS and USGS are detailed in Cross, Naugle, Carlson, and Schwartz (2016); Cross et al. (2017) and Row et al. (2015).

To ensure correct genotyping from low‐quality and low‐quantity feather DNA samples, each sample was PCR‐amplified twice across the 15 variable microsatellite loci to screen for allele dropout, stutter artifacts, and false alleles (DeWoody, Nason, & Hipkins, 2006). To minimize genotyping error, at least two independent observers scored each sample. If any locus failed to amplify in either replicate or if there was a discrepancy between locus genotypes as scored by the two observers, PCR amplification and genotyping were repeated twice more. If a genotype was confirmed by this repeat analysis, then it was retained. If a genotype failed again, the sample was assigned a missing score at the failed locus. To ensure consistency among laboratories, both laboratories genotyped the same 70 individuals (approximately 1% of the retained genotypes). Each laboratory's genotypes for these individuals were compared, and necessary shifts to synchronize allele calls were made for all samples.

To screen samples for quality control, we removed from analysis any individual for which amplification failed at one‐third of the loci (i.e., five loci). After removal of poor‐quality samples, genotypes were screened to ensure consistency between allele length and length of the microsatellite repeat motif using MICROCHECKER v2.2.3 (Van Oosterhout, Hutchinson, Wills, & Shipley, 2004). To identify and remove multiple captures of the same individual and to screen for and correct genotyping error, we used DROPOUT 2.3 (McKelvey & Schwartz, 2005 as implemented in Schwartz et al. 2006), MICROCHECKER v2.2.3 (Van Oosterhout et al., 2004), and package ALLELEMATCH 2.5 (Galpern, Manseau, Hettinga, Smith, & Wilson, 2012) in program R 3.3.0 (R Core Team 2016). Finally, we quantified the power of our microsatellite locus panel to discern individuals using probability identity (P_ID_; Evett & Weir, 1998) which calculates the probability that two individuals drawn at random from the population have the same genotype across all loci.

### 2.4 Network construction

A minimum of four or more individuals per node is required to calculate within‐node genetic variation (Dyer, 2014b). Therefore, before constructing the network, we performed a hierarchical clustering analysis of lek locations. First, we calculated a distance‐based tree using the geographic coordinates for the leks from which the 6,242 individual samples were collected (using the HCLUST function in base R). Second, we clustered all lek locations within 15 km of one another (cut distance implemented using the CUTREE function in base R). We selected 15 km as the cut distance, as this is the best estimate of median breeding dispersal distance among leks for sage‐grouse (Cross et al., 2017). Third, we removed any resultant clusters of leks composed of fewer than four individuals.

Following clustering, we constructed a weighted population network among the resulting clusters, which we henceforth refer to as nodes. For all clustered samples and for all nodes, we calculated the mean and standard deviation for number of alleles per locus, effective number of alleles, expected and observed heterozygosity, and *F*_IS_. We estimated genetic covariance among nodes, where microsatellite genetic covariance represents the weight of each network edge connecting nodes. We used the packages GSTUDIO (Dyer, 2014a) and POPGRAPH (Dyer, 2014a) in program R to estimate the conditional genetic covariance network following the methods of Dyer and Nason (2004) using default parameters (α = 0.05 and tolerance = 1 × 10^−4^; Garroway et al., 2008). Following pruning using the recommended settings, the resultant minimal incidence matrix contained the smallest set of edges that sufficiently capture the among‐node genetic covariance structure (Dyer & Nason, 2004). We also calculated the minimum spanning tree, which is the subset of network edges that connect all nodes together with the maximum genetic covariance among nodes (edge weight), without any cycles. To test for structure within the minimum spanning tree, we tested for correlation between weighted (factoring genetic covariance) distances among nodes in the minimum spanning tree and geographic distance (great circle distance) among all nodes calculated using the RDIST.EARTH function in the FIELDS package (Nychka, Furrer, Paige, & Sain, 2015) in R.

### 2.5 Network structure determination

To determine the network structure, we compared the degree distribution, clustering coefficient, and characteristic path length of the sage‐grouse genetic network to that of 1000 Erdos–Renyi model random networks with the same number of nodes, edges, and edge weight distribution as the range‐wide sage‐grouse genetic network. The characteristic path length is defined as the average shortest path length between all pairs of nodes in the network, and it provides an understanding of how long it takes alleles to traverse the network. We generated the random networks using package IGRAPH (Csardi & Nepusz, 2006) in program R and tested for significant differences between the degree distribution, clustering coefficient, and characteristic path length of the true sage‐grouse network and the random networks using permutation tests, following the methods of Garroway et al. (2008). We used the results of these comparisons to determine whether network structure was purely a function of the number of nodes and edges or whether network structure was a result of nonrandom processes. For example, if we found a characteristic path length that did not deviate significantly from that of the random networks coupled with a significantly higher clustering coefficient than that of the random networks, then we could conclude that the network had small‐world or scale‐free characteristics (Watts & Strogatz, 1998). Furthermore, if we found a degree distribution that did not follow the power law (which would indicate scale‐free network), was not binomial (which would indicate a random network) or fixed (which would indicate a regular network), but instead that was fat‐tailed, we would conclude that the likely network structure was that of the hub node‐and‐spoke small‐world network.

We quantified pairwise conditional genetic distance among all nodes. Conditional genetic distance is the length of the shortest path connecting each pair of nodes conditioned on network structure (Dyer, Nason, & Garrick, 2010) or the relative strength of the genetic covariance between nodes along the connecting edges (Koen, Bowman, Garroway, & Wilson, 2013). When compared to geographic distance among nodes, conditional genetic distance can provide insight into network structure. For example, if conditional genetic distance is correlated with geographic distance, one can conclude that the process of isolation by distance shaped a genetic network (Dyer et al., 2010).

We calculated six centrality indices (Table 1) and used these metrics to quantify connectivity and relative isolation of each node across the network. To calculate standard error (SE) of the mean and median as well as their respective 95% confidence intervals (CI), we calculated 1000 resampled networks of 75% of the nodes. Betweenness centrality quantifies the importance of a node in terms of the bottleneck to gene flow it creates, eigenvector centrality quantifies how connected a node is, and strength quantifies how strong the connection is between a node and all its neighboring nodes (Garroway et al., 2008). Eigenvector centrality is an index of both how well a node is connected and how well a node's immediate connections are connected—in essence, measuring both direct and indirect connectivity. These properties make eigenvector a better index than betweenness if one is interested in quantifying the strength of connections. This is because eigenvector centrality increases not only just with increased immediate connectivity of the node of interest, but also the immediate connectivity of the nodes to which the node of interest is connected. When quantifying connectivity, we used the centrality index of node strength rather than node degree, as Koen et al. (2015) found that strength more adequately depicts migration and gene flow than degree centrality. To examine relationships between network centrality indices, we tested for pairwise correlation between all indices using Spearman's rank correlation. We also tested for correlation between each network centrality index and mean peak male count per node (calculation described below). All network centrality indices were calculated using the IGRAPH package in R, and all correlations were calculated in base R.

To identify hub nodes of genetic exchange, we screened all nodes within the top 1% of each network centrality index (Table 1), and within the top 50% of all node centrality indices combined in order to identify those nodes that were most important to local network (regional) and network‐wide (range‐wide) connectivity. These nodes represent the top hub nodes of genetic exchange that maintain connectivity at all scales. To identify spoke nodes, we identified the nodes with the lowest ranking for each centrality index.

### 2.6 Keystone nodes

We hypothesized that nodes composed of the most highly attended leks and the most geographically central nodes in the species’ range would rank highest for centrality (i.e., node abundance and range centrality would be positively correlated with node centrality). To calculate abundance, we used the per‐lek high male counts recorded between 2005 and 2015 (WAFWA, 2015) and calculated the mean peak male count per node over these years using all leks constituting each node (male lek attendance is significantly correlated with female lek attendance; Bradbury et al., 1989). Using mean peak male count, we tested for correlation with network centrality indices.

We defined range centrality as the great circle distance from the center of the geographic center of the sampling distribution. We calculated range centrality as the distance of each node from the centroid of a minimum convex polygon enveloping all nodes, such that increased magnitude of distance was equivalent to inverse range centrality. We calculated the minimum convex polygon using the GCONVEXHULL function and calculated the centroid of the minimum convex polygon using the GCENTROID function in the RGEOS package (Bivand & Rundel, 2017) in R. Finally, we calculated the distance of every node from the centroid using the RDIST.EARTH function in the FIELDS package (Nychka et al., 2015) in R. Using range centrality, we tested for correlation with node centrality indices.

Finally, we sought to identify nodes with greater importance to genetic connectivity than the magnitude of lek attendance within the node or node location within the species range alone might indicate. We call these nodes keystone nodes. We identified keystone nodes as those that were low in attendance or peripheral to the range, but that still ranked high in centrality. To identify keystone nodes, we plotted both the mean peak male count within a node and the range centrality of each node against each network centrality index. We then called the outliers of these plots, keystone nodes.

## 3 RESULTS

### 3.1 Genotyping and network construction

After genotyping the same 70 individuals at each laboratory, we compared allele calls. For two loci, the laboratories had a two base pair difference across all allele calls (BG6 and SGCA5), for one locus, there was a four base pair difference (SGCA11), and for another locus, a seven base pair difference (SGCTAT1) as well as two alleles called off‐step on a dimer repeat motif changed to comply with the tetramer repeat motif. Each laboratory's genotypes for these individuals were shifted to synchronize allele calls for all samples for these loci. With the additional ALLELEMATCH analysis, we discovered no additional multiple matches (identical genotypes resulting from the same sample being genotyped by both laboratories). P_ID_ for the complete microsatellite panel was 2.20 × 10^‐22^, providing evidence that our microsatellite panel was adequate for distinguishing individuals. Our genotyping efforts resulted in 6,729 individual genotypes from 1,388 leks (median = 3 individuals per lek, IQR = 4 individuals per lek, range = 1–62 individuals per lek) following duplicate removal and quality control.

**Figure 2:**
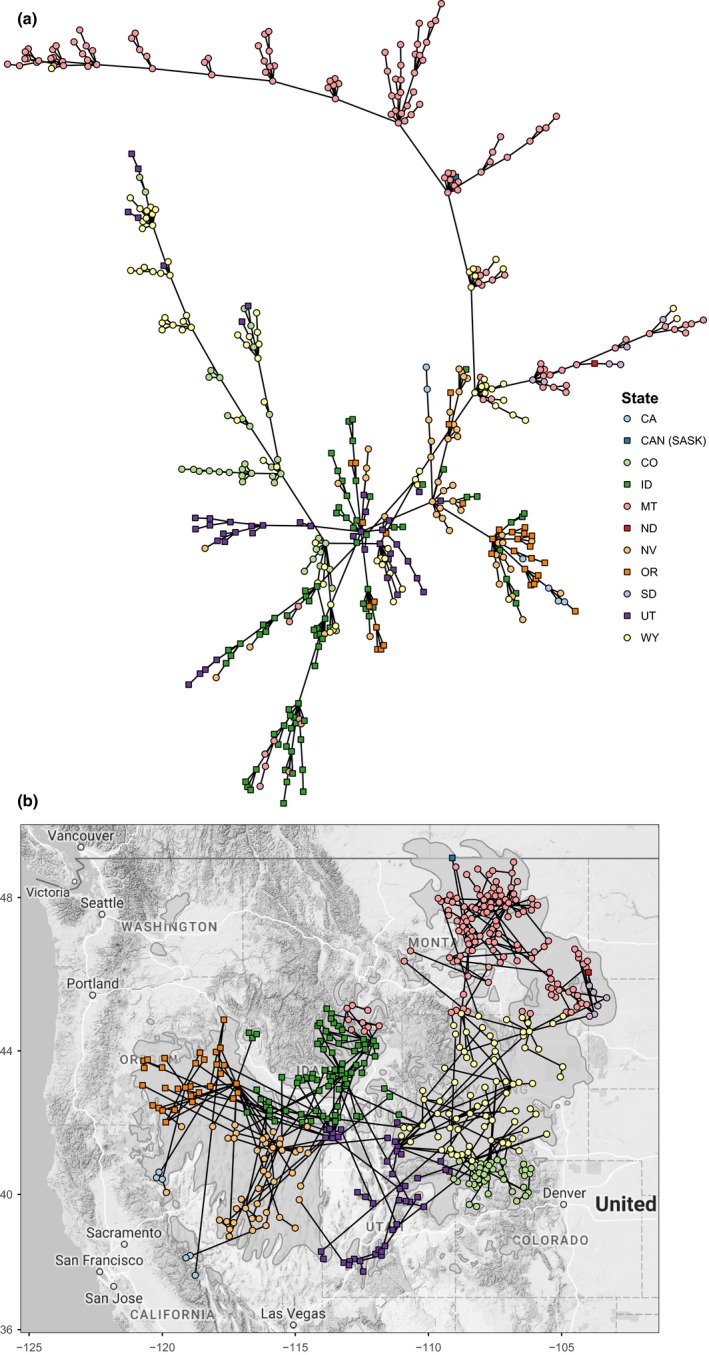
The greater sage‐grouse range‐wide genetic network minimum spanning tree. The minimum spanning tree is pruned such that only the most highly weighted edges (i.e., the connections representative of the greatest genetic covariance) are shown between all nodes (*n *=* *458). Distance among nodes in the minimum spanning tree was highly correlated with geographic distance between nodes (*r*_*s*_ = 0.61, *p *<* *2.2 × 10^−16^). (a) Fruchterman‐Reingold plot (layout with minimal edge overlap). (b) Geographic map of the range‐wide greater sage‐grouse genetic network nodes connected by edges retained within the minimum spanning tree. Node color indicates geographic location by state. Edges are shown as black lines. The species’ range is shown as light gray polygons

Hierarchical clustering and removal of nodes with fewer than four individuals yielded 5,924 samples from 1,180 leks clustered into 458 nodes from 2006 to 2015 (median = 10 individuals per node, IQR = 7.00–15.75, range = 4–90 individuals per node, Table 2). We were able to use 1,057 mean peak male counts for 399 nodes. For all clustered samples and across all nodes, average number of alleles was 17.0 ± 7.48 (mean ± SD) and 6.43 ± 1.47, for the effective number of alleles was 7.30 ± 2.88 and 4.41 ± 0.75, for expected heterozygosity was 0.84 ± 0.06 and 0.74 ± 0.05, for observed heterozygosity was 0.78 ± 0.05, and for *F*_IS_ was 0.07 ± 0.02 and −0.06 ± 0.06. We constructed a network composed of 458 nodes connected by 14,481 edges. The minimum spanning tree (Figure 2a) was correlated with geographic distance (*r*_*s*_ = 0.61, *p *<* *2.2 × 10^−16^). Montana nodes compose a large group within the minimum spanning tree and are joined to the rest of the network through nodes in Wyoming and Idaho (Fig 2). Nodes in Colorado also group together and are linked by nodes in Wyoming and Utah (Figure 2). There was strong evidence for a positive correlation between conditional genetic distance and geographic distance (Table 4).

**Table 2:** Sample summary listed by U.S. state or Canadian province for all samples used to construct range‐wide greater sage‐grouse genetic network. Total individuals sampled per state/province, leks sampled per state/province, and total number of nodes per state/province

State/Province	Individuals	Leks sampled	Nodes
CA	53	14	6
CAN (SASK)	6	2	1
CO	679	106	38
ID	988	281	80
MT	1881	358	130
ND	7	2	1
NV	430	116	45
OR	296	52	31
SD	75	15	6
UT	607	114	44
WY	902	120	76

### 3.2 Network structure determination

The sage‐grouse genetic network deviated from random network structure in both mean clustering coefficient and mean characteristic path length. Both indices were significantly greater for the sage‐grouse genetic network than for the 1000 random networks with the same number of nodes, edges, and edge weight distribution as the sage‐grouse network; none of the random networks had a greater mean clustering coefficient or characteristic path length than the sage‐grouse network (clustering coefficient: *p *<* *.001, characteristic path length: *p *<* *.001). Both the mean clustering coefficient (0.19 ± 3.35 × 10^−3^ [SE]) and the mean characteristic path length (1.88 ± 7.04 × 10^−3^ [SE], [1.88, 1.91]) were short, and the node degree distribution was fat‐tailed (Figure 4d).

### 3.3 Node properties

**Figure 3:**
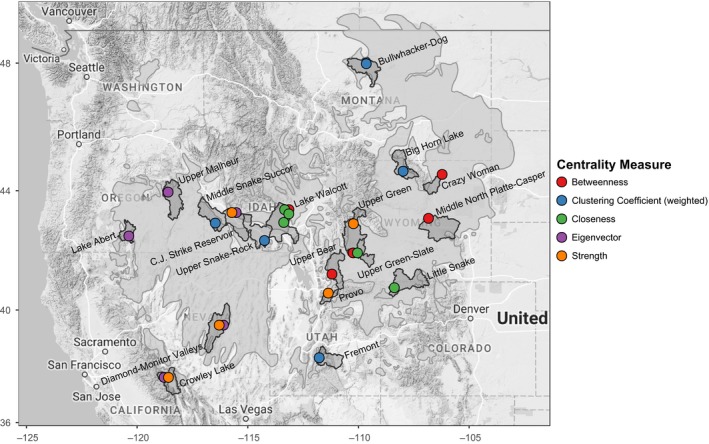
The top 1% of nodes for each of the six centrality indices (*n *=* *20 per index). Nodes in the top 1% of more than one index are offset to the left or right to reveal both. Node color indicates centrality measure. Shaded polygons depict the watershed within which these top‐ranking nodes are located. The species’ range is shown as light gray polygons

In order to describe node location, we used USGS hydrologic cataloging units, also known as watersheds (https://water.usgs.gov/GIS/metadata/usgswrd/XML/huc250k.xml). We discovered hub nodes within the C.J. Strike Reservoir watershed in Idaho and the Big Horn Lake and Upper Green‐Slate watersheds in Wyoming. These nodes rank highly across multiple centrality indices indicating the importance of these regions to maintaining genetic connectivity across the network. Collectively, these two basins contained nodes with the maximum centrality indices (Figure 3). Many other regions contained hub nodes as determined with one or more centrality indices. Notably, the Big Horn Lake watershed in Wyoming, the Bullwhacker‐Dog and Middle Musselshell watersheds in Montana, the Fremont watershed in Utah, and the Middle Snake‐Succor watershed in Idaho and Oregon contain nodes that rank high for centrality indices indicative of their functioning as hub nodes that maintain local connectivity. The Crazy Woman watershed, the Upper Green‐Slate watershed, and the Middle North Platte‐Casper watershed in Wyoming, the Lake Walcott watershed in Idaho, and the Upper Bear watershed in Utah all contain nodes that rank high for centrality indices indicative of their functioning as hub nodes that maintain network‐wide connectivity (Figure 3).

Only five nodes ranked in the top 50th percentile of all network centrality indices, indicating their importance to genetic connectivity both locally and network‐wide as well as the rarity of this combination of local and network‐wide importance. The range of each centrality index for these hub nodes was large (Table 3). These nodes were located within the Idaho Falls, Lake Walcott, and Salmon Falls watersheds in Idaho, and the Middle North Platte‐Casper and Sweetwater watersheds in Wyoming (Figure 3); locations central to both the sampling extent and the geographic range of the species.

**Table 3:** Network centrality indices (betweenness, closeness, clustering coefficient, degree, and eigenvector) and network connectivity (strength and weight) for the range‐wide greater sage‐grouse genetic network (a) and networks (b) calculated from 1000 networks constructed from a resample of 75% (*n *=* *343 nodes) of the originally sampled 458 nodes (sampled without replacement). Listed are the network centrality index, the component for which each index was calculated, minimum, mean, median, standard error (SE) of the mean and median, and 95% confidence intervals (CI) of the mean and median

(a)					
Centrality index	Component	Min	Mean ± SD	Median (IQR)	Max
Betweenness centrality	Node	0.00	203.60 ± 249.63	90.50 (29.25–270.50)	1491.00
Closeness centrality	Node	4.91 × 10^−5^	1.34 × 10^−4^ ± 1.51 × 10^−5^	1.37 × 10^−4^ (1.28 × 10^−4^–1.45 × 10^−4^)	1.59 × 10^−4^
Clustering coefficient	Node	0.12	0.19 ± 0.022	0.18 (0.17–0.20)	0.33
Eigenvector centrality	Node	0.07	0.55 ± 0.18	0.57 (0.43–0.69)	1.00
Strength	Node	88.19	619.10 ± 181.51	634.70 (488.60–752.50)	1085.00
Weight	Edge	3.02	9.82 ± 2.23	9.68 (8.42–11.00)	35.61

(b)			
Centrality index	Component	Resampled networks	
		Mean ± SE [95% CI]	Median ± SE [95% CI]
Betweenness centrality	Node	155.77 ± 1.28 [153.28, 158.26]	87.00 ± 4.87 [78.00, 98.00]
Closeness centrality	Node	1.75 × 10^‐4^ ± 1.83 × 10^‐6^ [1.71 × 10^‐4^, 1.78 × 10^‐4^]	1.78 × 10^‐4^ ± 1.87 × 10^‐6^ [1.75 × 10^‐4^, 1.82 × 10^‐4^]
Clustering coefficient	Node	0.18 ± 0.0032 [0.18, 0.19]	0.18 ± 0.0032 [0.17, 0.19]
Eigenvector centrality	Node	0.55 ± 0.025 [0.49, 0.59]	0.56 ± 0.027 [0.50, 0.60]
Strength	Node	449.46 ± 8.82 [431.19, 466.40]	455.71 ± 10.22 [435.06, 475.58]
Weight	Edge	9.86 ± 0.066 [9.74, 9.99]	9.74 ± 0.069 [9.60, 9.87]

**Figure 4:**
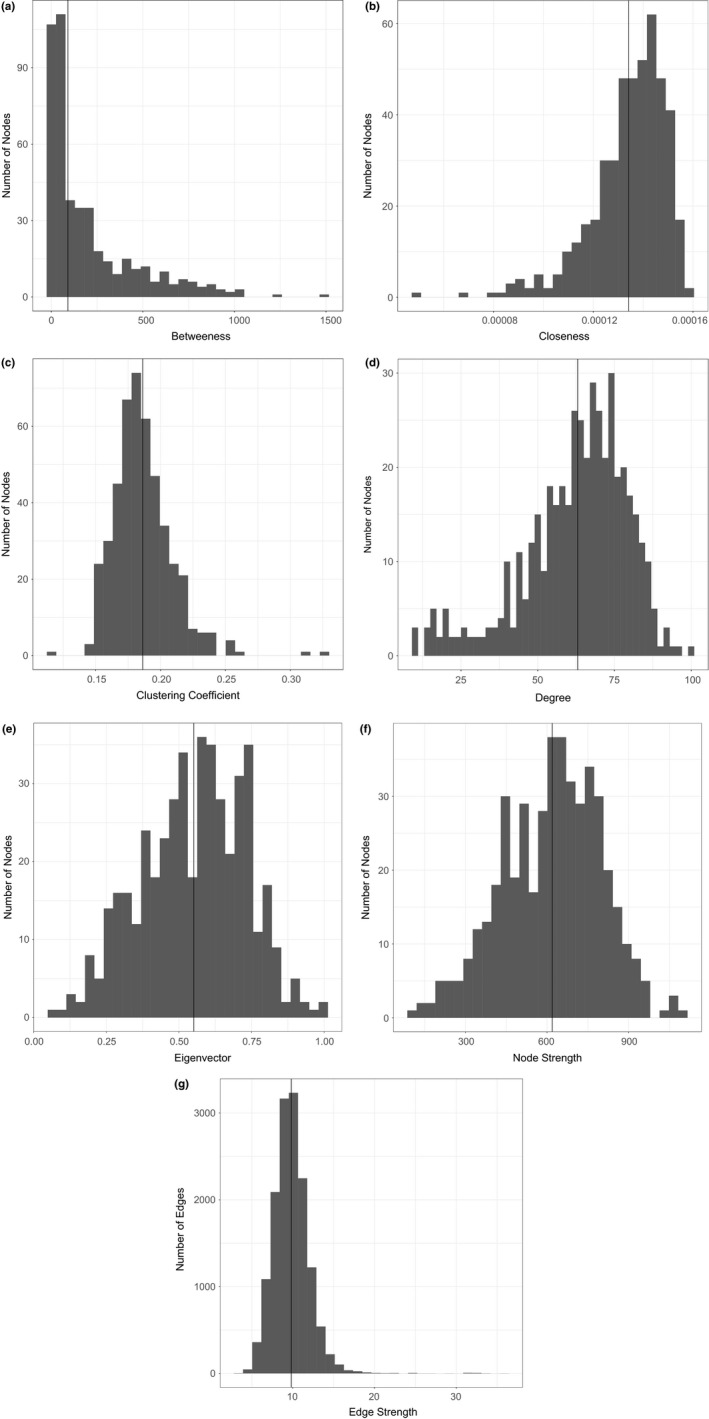
Centrality index distributions for all nodes (a–f; *n *=* *458) and edges (g; *n *=* *14,433) in the greater sage‐grouse genetic network. The solid vertical black line shows the median for each index

Nodes with high betweenness centrality act as bridges between different parts of the network, so their loss can have network‐wide impacts on genetic connectivity (Garroway et al., 2008). We identified several hub nodes whose betweenness ranking was high, located toward the center of the species’ range. Three of the top 1% of nodes ranked by betweenness centrality were located in Wyoming—one in the Crazy Woman watershed, one in the Middle North Platte‐Casper, and one in the Upper Green‐Slate watershed. Of the remaining two nodes, one was located in the Lake Walcott watershed in Idaho, and one was located in the Upper Bear watershed in Utah (Figure 3). Of these, the node with the greatest betweenness (1491) was located just south of Grand Teton National Park in the Upper Green‐Slate watershed. This same node also indexed high for strength (700.89). Most nodes in the network were important to network‐wide connectivity (right‐skewed distribution: Table 3, Figure 4). However, seventeen nodes had a betweenness of zero, indicating relatively low importance to fostering genetic connectivity across the network. These spoke nodes had an average strength of 429.2 (±218.3 [SD]), indicating strong connections to other nodes despite low importance to network‐wide genetic connectivity. Many of these spoke nodes with the lowest betweenness indices were located toward the periphery of the species’ range.

To identify nodes that covaried the greatest with all other nodes in the network, we ranked nodes by closeness centrality. Closeness is an index of the average shortest path between a node and all other nodes in the network. Hence, a smaller closeness index indicates shorter paths on average, and therefore, greater connectivity. There were a small number of very closely covarying nodes in the network (left‐skewed distribution: Table 3, Figure 4). The top‐ranked closeness nodes were central to the species’ range, away from the periphery. Two of the nodes in the top 1% of closeness centrality were located in the Lake Walcott watershed in Idaho. The remaining nodes were located in the Little Snake watershed in Colorado and the Sweetwater and Upper Green‐Slate watersheds in Wyoming (Figure 3). The node with the minimum closeness (4.91 × 10^−5^) was located in the Spring‐Steptoe Valleys watershed in Nevada and had low betweenness (0). The node with the greatest closeness in the network (1.59 × 10^−4^) was located just south of Grand Teton National Park in the Upper Green‐Slate watershed in Wyoming and had a relatively high betweenness (1521) and strength (700.89).

To identify nodes that anchor tightly knit groups connected by a high number of edges, we examined node rankings by clustering coefficient. Increased clustering coefficient is indicative of small‐world characteristics. Network‐wide, there was a low chance that any two nodes connected to a given node were also connected to one another (right‐skewed distribution: Table 3, Figure 4). The nodes in the top 1% of clustering coefficient were found across the species’ range and were mostly toward its periphery (Figure 3). The southernmost hub node in the top 1% was in the Fremont watershed in Utah. Other hub nodes in the top 1% of centrality were located in the Middle Snake‐Succor watershed in Idaho, the Big Horn Lake watershed in Wyoming, and the Bullwhacker‐Dog watershed and Middle Musselshell watershed in Montana. The hub node with the greatest clustering coefficient (0.33) was found in the Middle Snake‐Succor watershed. This node also had low betweenness (0), strength (223.65), and eigenvector centrality (0.21). The spoke node with the lowest clustering coefficient (0.12) was found in the Shoshone watershed in northcentral Wyoming and had low betweenness (7), strength (327.34), and eigenvector centrality (0.25).

To identify the most highly networked nodes, we examined node rankings by eigenvector centrality. Eigenvector centrality increases for nodes that are highly connected to other highly connected nodes. All but one of the top one percent of nodes ranked by eigenvector centrality were located in the Great Basin, indicating increased genetic connectivity therein. These hub nodes are located in the Crowley Lake watershed in California, the Lake Abert watershed in Oregon, the Diamond‐Monitor Valleys watershed in Nevada, and the Upper Malheur watershed in Oregon (Figure 3). The exception was a single node outside the Great Basin in the C.J. Strike Reservoir watershed in Idaho. This hub node had the greatest eigenvector centrality (1.00) and had very high strength (1064.07), but very low betweenness centrality (3). The spoke node with the lowest eigenvector centrality (0.067) was located in the Escalante Desert watershed in Utah and was a terminal node on the minimum spanning tree. This spoke node was also low for strength (88.2) and betweenness (69): low centrality both locally and network‐wide. Eigenvector centrality was normally distributed (Table 3, Figure 4).

To determine which nodes covaried closely with many other nodes, we calculated the strength of each node. The top 1% of nodes ranked by strength was located toward the range periphery in both the central and southwestern part of the species’ range, indicating increased genetic covariance among a greater number of nodes (Figure 3). Of the hub nodes with the greatest strength, one hub node was in the Crowley Lake watershed in California, one in the Diamond‐Monitor Valleys watershed in Nevada, one in the C.J. Reservoir Strike watershed in Idaho, one in the Provo watershed in Utah, and one in the Upper Green watershed in Wyoming. All five of the top 1% of nodes by strength were terminal nodes on the minimum spanning tree. The nodes with the greatest and least strength (1085.0 and 88.2), located in the C.J. Strike Reservoir watershed in Idaho and the Escalante Desert watershed in Utah, were also similarly ranked for eigenvector centrality (two indices for which there was very strong evidence for a positive correlation (Table 4)). Node strength was normally distributed (Table 3, Figure 4).

**Table 4:** Correlation between network centrality indices, range centrality, and lek attendance per node for the range‐wide greater sage‐grouse genetic network. Spearman's rank correlation (*r*_*s*_) is shown below the diagonal with significance (*p*) above

	A	He	Betweenness	Closeness	Clustering coefficient	Eigenvector	Strength	Samples in cluster	Overall mean peak male count	Range centrality (inverse)
A	―	0.000	0.000	0.000	0.000	0.000	0.000	0.000	0.002	0.000
He	0.872	―	0.000	0.000	0.000	0.000	0.000	0.000	0.028	0.000
Betweenness	0.706	0.565	―	0.000	0.749	0.000	0.000	0.000	0.028	0.237
Closeness	0.670	0.595	0.914	―	0.380	0.008	0.007	0.000	0.046	0.005
Clustering coefficient	0.341	0.278	−0.015	−0.041	―	0.000	0.000	0.000	0.314	0.429
Eigenvector	−0.515	−0.307	−0.280	−0.124	−0.499	―	0.000	0.000	0.003	0.000
Strength	−0.551	−0.359	−0.281	−0.125	−0.567	0.983	―	0.000	0.006	0.002
Samples in cluster	0.860	0.602	0.665	0.547	0.340	−0.577	−0.613	―	0.021	0.310
Overall mean peak male count	−0.157	−0.110	−0.110	−0.100	−0.051	0.149	0.137	−0.116	―	0.449
Range centrality (inverse)	−0.234	−0.388	−0.055	−0.131	0.037	−0.206	−0.147	−0.048	0.038	―

We found evidence for a strongly positive significant correlation between betweenness and closeness, and eigenvector and strength (Table 4). All other significant correlations among centrality indices were weak and negative. We found evidence for a strongly positive significant correlation between number of alleles per node and betweenness and closeness, although all other significant correlations were moderately negative or weak and positive. The evidence for a correlation between mean peak male count and centrality indices was weak when significant. Finally, we found evidence for a strongly positive significant correlation between the number of samples in a node and betweenness, but only moderate or weak relationships when testing for correlation with other centrality indices.

### 3.4 Edge properties

Edge weight is an index of the magnitude of genetic covariance between nodes and can be used to identify nodes most closely linked. Overall, genetic connectivity among nodes has led to increased network connectivity, with the occurrence of some highly connected nodes evidenced by a skewed right distribution of edge weight (Table 3; Figure 4). The top 0.1% of edges with the greatest genetic covariance emanated from a node in the Spring‐Steptoe Valleys watershed in Nevada. This node also has the lowest closeness centrality and low eigenvector centrality (0.33) and very low betweenness (0). The edge of least weight connected two nodes within the Fremont watershed in Utah (in the southcentral UT group of nodes in Figure 3). This node was of moderate importance to network‐wide connectivity (betweenness: 115), but had low connectivity to other nearby nodes (eigenvector centrality: 0.11).

### 3.5 Keystone nodes

**Figure 5:**
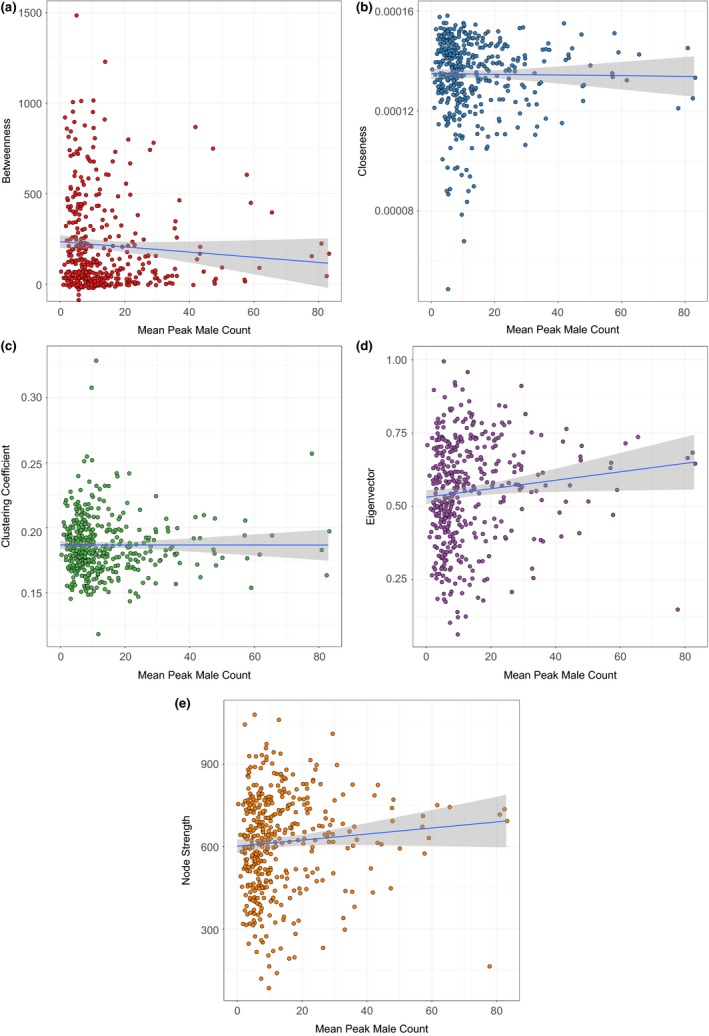
Relationships between centrality index (*y*‐axis) and mean peak male count per node (*x*‐axis). Red circles envelope keystone nodes. The fitted linear model and confidence interval are shown (blue line with shaded confidence interval)

It was common that the hub nodes—those with the highest centrality rankings—were also those with lower mean peak male count (Figure 5). There was strong evidence for a weak positive correlation between mean peak male count and eigenvector centrality and mean peak male count and strength and strong evidence for weak negative correlation between mean peak male count and betweenness and between mean peak male count and closeness (Table 4 and Figure 5).

**Figure 6:**
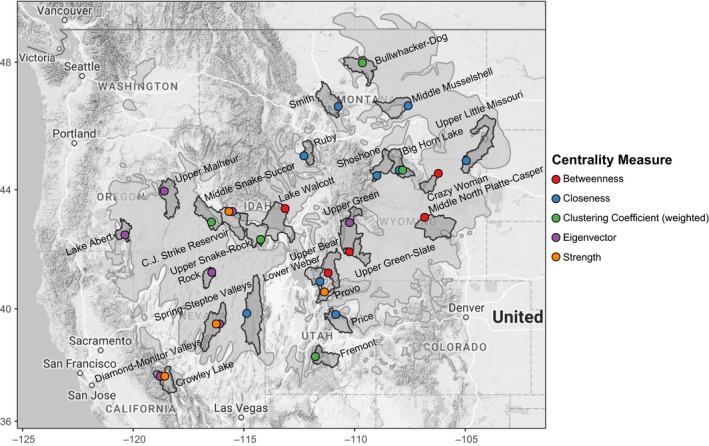
Keystone nodes (*n *=* *26): nodes with greater importance to genetic connectivity than the magnitude of lek attendance within the node or node location within the species range alone might indicate. These nodes were low in mean peak high male count relative to their network centrality rankings. Points representing keystone nodes for more than one centrality index are offset to the left or right, such that these offset touching points represent the same node. Node color indicates centrality measure. Shaded polygons depict the watershed within which these top‐ranking nodes are located. The species’ range is shown as light gray polygons

Across all centrality indices, we discovered 26 nodes that ranked high for network centrality despite having lower mean peak male count than nodes of similar ranking (Figure 6). These 26 keystone nodes were located across the entire species’ range. Four of these keystone nodes ranked highly for more than one centrality index, with high rankings coupled for eigenvector centrality and strength and for closeness and clustering coefficient. In all cases, these nodes were keystone for betweenness and closeness or for eigenvector and strength.

## 4 DISCUSSION

### 4.1 Emergent network properties

The greatest utility of our network analysis is its ability to be used to prioritize and target conservation efforts to the nodes most important to maintaining network connectivity at any desired scale. Our network approach allows nodes to be ranked across multiple centrality indices, indicative of different scales and patterns of connectivity, each with unique importance to conservation.

We discovered that the sage‐grouse range‐wide genetic network is best characterized as hub‐and‐spoke topology most resembling the structure of a small‐world network and not that of a random or regular network. Both the mean clustering coefficient (0.19 ± 3.35 × 10^−3^ [SE]) and the mean characteristic path length (1.88 ± 7.04 × 10^−3^ [SE], [1.88, 1.91]) were shorter than has been reported for other species (e.g., 0.254 and 2.26 in Garroway et al., 2008). The fat‐tailed distribution of node degree (Figure 4d) confirmed small‐world network structure by ruling out scale‐free structure, for which the degree distribution follows a power law.

Many hub nodes of connectivity within the network are located across the species’ range (Figure 3), with most spoke nodes located along the periphery of the range. This hub‐and‐spoke topology is evident in the minimum spanning tree, with important hub nodes of genetic connectivity occurring in nearly every state across the contiguous range (Figure 2). Loss of one of these highly connected hub nodes within several major basins could severely affect overall network connectivity.

We documented strong connectivity across the entire network, evidenced by high‐ranking nodes and edges across the species’ range. This means that some of the nodes may be able to recover should they be extirpated but the habitat remain intact or be restored (e.g., following a local extinction caused by West Nile virus—e.g., Naugle et al., 2004; or return after restoration following natural resource extraction—e.g., Naugle et al., 2011). The ability to recover is exhibited in the network's traversability (i.e., the apparent low resistance to gene flow). However, to the best of our knowledge, node recovery has not been previously investigated in wildlife networks. The minimum spanning tree can serve as a powerful guide in making management decisions related to the relative importance of individual nodes to overall landscape connectivity (Urban & Keitt, 2001), as it is possible to model which nodes or which parts of the range will most likely be affected by the loss of any given node.

Our results suggest that distance plays an important role in structuring genetic connectivity (also known as isolation by distance, Wright, 1943). The vast majority of edges in the minimum spanning tree—those connections that represent the greatest covariance—connect geographically proximal nodes (Figure 2a). Similarly, there was a correlation between conditional genetic distance and geographic distance. These results support prior findings of isolation by distance across the species’ range (Bush et al., 2011; Cross et al., 2016; Davis, Reese, Gardner, & Bird, 2015; Fedy, Row, & Oyler‐McCance, 2017; Oyler‐McCance, Taylor, & Quinn, 2005; Schulwitz, Bedrosian, & Johnson, 2014). However, nodes with greater centrality, important to both local and network‐wide genetic exchange, are located across the species’ range.

Both Cross et al. (2016) and Oyler‐McCance et al. (2005) found that sage‐grouse subpopulations in southwestern Montana were diverged from populations in the rest of the state. We confirm this prior finding, showing that sage‐grouse from these same subpopulations are more closely related to conspecifics in Idaho than to subpopulations in Montana, as is evident in edge connectivity within the minimum spanning tree (Figure 2b). Cross et al. (2016) also found that the population in Northern Montana was diverged from the subpopulation in Southeastern Montana and the Dakotas and from the southcentral Montana subpopulation (the SE‐W subpopulation in Cross et al., 2016). We confirm these findings here, showing nodes with very high clustering coefficient (indicative of highly interconnected network subunits) within the same regions (Figure 3). We expect that the other top‐ranked nodes for clustering coefficient in the Middle Snake‐Succor watershed in Idaho and the Fremont watershed in Utah might also be embedded at the core of their respective subpopulations. Schulwitz et al. (2014) found that the subpopulations in southern and southeastern Montana and the Dakotas were both highly connected to leks in northern Wyoming. We also found the same pattern of connectivity, evident in the hub‐and‐spoke topology of the minimum spanning tree. In our case, a hub node in Wyoming/southcentral Montana is located in the Big Horn Lake watershed of northcentral Wyoming, and a hub node for Wyoming/southeastern Montana subpopulations is located within the Crazy Woman watershed of northeastern Wyoming (Figures 2b and 3). Davis et al. (2015) found that the small northern California population known to have experienced population declines had retained genetic diversity. We confirm this understanding by finding that the nodes in this area show elevated local connectivity (covariance) within the area. We also found that genetic connectivity into the northern California nodes comes from nodes to the north in Oregon (Figure 2b). Oyler‐McCance, Casazza, Fike, and Coates (2014) discovered a northern and a southern subpopulation within the Bi‐State population in southern California and southwestern Nevada. We found the same break evidenced by a lack of edges connecting these two units in the minimum spanning tree (Figure 2b). This lack of interconnectivity among nodes in the northern and southern groups is especially surprising, given that both groups exhibit greater covariance with far more geographically distant nodes. Fedy et al. (2017) documented genetic differentiation between birds in the Bighorn and Powder River Basins of Wyoming as well as differentiation between the northern and southern parts of the state, differences reflected in our analysis as evidenced by edge connectivity within the minimum spanning tree.

### 4.2 Hubs of genetic exchange

We identified nodes with high importance to large‐scale, network‐wide genetic connectivity (i.e., nodes with high betweenness), and nodes within the top 50% of all centrality indices important to both network‐wide and local connectivity. These top‐ranked hub nodes are located across the entire range of the species. The locations of these hub nodes important to network‐wide connectivity are in areas that should foster range‐wide genetic connectivity due to their location in the topography of the western landscape.

Connectivity of these hubs is apparent in the minimum spanning tree (Figure 2b) where connectivity across the range appears crescent shaped, with one point of the crescent in northern Montana/Saskatchewan and the other in Oregon. The Upper Snake Basin of Idaho (Lake Walcott watershed) forms a thumb terminating in southwestern Montana to the northeast. Hub connectivity opens up over the Columbia Plateau of Idaho (Upper Snake‐Rock, C.J. Strike Reservoir, and Middle Snake‐Succor watersheds). Connectivity extends south into the Great Basin composing most of eastern California, all of Nevada and western Utah. Here, the Southwest River Basin in Idaho (Middle Snake‐Succor and C.J. Strike Reservoir watersheds) connects to the Malheur Basin (Upper Malheur watershed) to the west and to the South Lahontan River Basin (Crowley Lake watershed) by way of the Central Nevada Desert (Diamond‐Monitor Valleys watershed) to the southwest. The Green River Basin of Wyoming (Upper Green and Upper Green‐Slate watersheds) sits just west of a low section of the North American Continental Divide connecting the Upper Snake Basin and Great Basin to the rest of Wyoming and farther up into the northeastern part of the species range. The Green River Basin also sits just north of the Yampa and White River Basins in Colorado (Little Snake watershed), and the Bear River Basin in Utah (Upper Bear watershed), both connected by low valleys to the south. Similarly, the Powder and Tongue River Basins of Wyoming (Crazy Woman watershed) connect to the north with both of the Dakotas and eastern Montana. The Bighorn River Basin, which ranks lower for other connectivity indices, connects to the southeastern‐west subpopulation in the Yellowstone River Basin of Montana (Cross et al., 2016)—nodes in the Big Horn Lake watershed anchor both basins. We suspect that the topology of the genetic network is largely shaped by the topography of the landscape, a hypothesis previously posited for sage‐grouse (Cross et al., 2016; Row et al., 2015; Schulwitz et al., 2014), and which has been found to influence genetic structure in other species (e.g., Roffler et al., 2014).

We identified 26 keystone nodes across the range of sage‐grouse that stand out with increased importance to genetic connectivity despite having lower mean peak male count (Figure 6). These keystone nodes do not follow the presupposition of increased centrality with increased mean peak male count (i.e., a proxy for population size for any given node) and include the highest ranking nodes for each centrality index, regardless of the population size (Figure 5). We believe that these keystone nodes and other hub nodes (Figure 3) are top candidates for targeted conservation efforts, as their protection will help secure range‐wide genetic connectivity. The keystone nodes are also distributed across the entire species’ range, from the core to the periphery (Figure 6). Therefore, neither range centrality nor local population size alone should be trusted proxies for prioritizing targeted conservation actions for sage‐grouse.

### 4.3 Limitations of the study and future directions

Prior research has modeled range‐wide sage‐grouse connectivity using a network approach. Knick and Hanser (2011) weighted nodes using lek attendance and limited edge connections using hypothesized dispersal thresholds. However, these imposed dispersal thresholds may have affected the resultant network structure. For example, Knick and Hanser (2011) used an exponential decay function to determine the probability of connectivity of leks. Imposing dispersal thresholds likely oversimplified the contribution that each priority area for conservation made to network connectivity by assuming dispersal limitations is equal among all nodes regardless of the internal population dynamics within nodes and environmental conditions within and among nodes. Crist, Knick, and Hanser (2017) used network approaches to generate several models of hypothesized connectivity among sage‐grouse priority areas for conservation, which are areas that protect larger leks (i.e., those with more males visible during breeding) and surrounding area. They characterized the centrality of each priority area for conservation and concluded that several subnetworks exist across the species’ range. However, in their analysis, patch size, shape, and boundary length all had an effect on the pattern of connectivity and centrality. Our analysis provides insight into genetic connectivity using centrality indices based solely on the species’ biology: the genetic covariance resulting from cumulative dispersal and breeding, a quantitative metric.

We have confidence in the cut distance we used to cluster leks into nodes, as it is empirically based on dispersal distances documented over a vast area, across multiple years, involving both sexes (Cross et al., 2017). Our clustering approach increased genetic variance within nodes, but also increased covariance among nodes (Dyer, 2015). Choice of cut distance depends on the desired scale of analysis for conservation and management application. We could have performed this analysis using individual leks. Doing so would have resulted in finer resolution for our results. However, it also would have resulted in fewer individuals per node, which would have limited our characterization of within‐node genetic variation. Furthermore, we would have had to cut many leks from our analysis due to the minimum node composition requirement of four individuals. By clustering leks into nodes, we were restricted to making statements about the connectivity of larger landscapes that extend beyond the size of an individual lek and which were potentially representative of leks unsampled within the same landscapes. Furthermore, our clustering approach reflects the biology of the species, as prior research has shown that both female and male sage‐grouse attend multiple leks within a breeding season (Cross et al., 2017; Dunn & Braun, 1985; Semple, Wayne, & Gibson, 2001).

We found evidence for correlation between some network centrality indices and samples per node and mean peak male count. However, when significant, these relationships were only moderate or weak in all but one case: that of betweenness and samples within a node (Table 4). Therefore, we do not believe that sample size drove the centrality of a node. Larger populations acting as hub nodes might be expected, as these highly populated hub nodes would be expected to house greater genetic diversity to be the sources of dispersers. However, as discussed above, the highest ranking nodes for each centrality index were never those with the greatest mean peak male count (Figure 5).

Future work should examine the effect of the spatial distribution of individuals composing nodes on the resultant network model structure. For example, constructing a genetic network where priority areas for conservation serve as nodes may help prioritize conservation based on existing management boundaries at a larger landscape scale. It is worth noting that if priority areas for conservation are treated as nodes, larger priority areas for conservation may score higher for centrality indices due to the within‐node proportion of the genetic covariance, which will increase centrality.

### 4.4 Applications and future directions

We believe that the greatest utility of our network analysis will be its use in prioritizing and targeting conservation efforts to the nodes most important to maintaining network connectivity. This network approach allows for the ranking of nodes by multiple centrality indices, indicative of different scales and different patterns of connectivity. These indices can be used to locate the top‐ranking nodes—and more importantly, the leks which compose those nodes—which can then be prioritized in accordance with management goals (Bottrill et al., 2008). If goals are to conserve hubs of genetic exchange that connect the greatest number of nodes range‐wide, then ranking nodes based on betweenness is most relevant. If goals are to conserve hubs of genetic exchange that connect immediate connections, then ranking nodes based on eigenvector centrality is most relevant. If goals are to conserve nodes that have the greatest genetic exchange with their immediate connections, then ranking nodes based on strength is most relevant. If goals are to conserve hubs of local connectivity, then ranking nodes based on closeness or clustering coefficient is most relevant.

Conservation actions may be targeted first toward the top‐ranking nodes, or managers may first choose to combine network centrality with economic cost before deciding where to act. We can imagine many additional ways in which network centrality may be combined with additional metrics to target conservation resources. Our hope is that the empirically based sage‐grouse genetic network we constructed will prove a useful tool to conservation planners.

## CONFLICT OF INTEREST

None declared.

## AUTHOR CONTRIBUTIONS

T.B.C. conceived the idea, analyzed the data, conducted the research, and wrote the paper. T.B.C. and M.K.S. developed and designed the methods. T.B.C. and S.J.O. generated the data. T.B.C., D.E.N., S.J.O., J.R.R. B.C.F, S.T.K, and M.K.S. substantially edited the paper.

## DATA ACCESSIBILITY

Sample microsatellite genotypes and node membership are available on USGS ScienceBase: https://doi.org/10.5066/f73n22pn.
